# The involvement of circulating CD69^+^ CD56^bright^ natural killer cells in weight loss before bariatric surgery: A retrospective cohort study

**DOI:** 10.1097/MD.0000000000034999

**Published:** 2023-10-13

**Authors:** Emi Chikuie, Yoshihiro Saeki, Kazuaki Tanabe, Hiroshi Ota, Yuka Tanaka, Hideki Ohdan

**Affiliations:** a Department of Gastroenterological and Transplant Surgery, Graduate School of Biomedical and Health Science, Hiroshima University, Hiroshima, Japan; b Division of Endoscopic Surgery, Hofu Institute of Gastroenterology, Hiroshima University Hospital, Hofu, Japan; c Department of Perioperative and Critical Care Management, Graduate School of Biomedical and Health Sciences, Hiroshima University, Hiroshima, Japan.

**Keywords:** bariatric surgery, natural killer cells, obesity, weight loss

## Abstract

As the impact of the immune system on weight loss prior to bariatric surgery has never been proven, we elucidated the clinical utility of the immune system as an indicator of preoperative weight loss before bariatric surgery. We examined the relationships between preoperative weight loss and biochemical and clinical data at the initial visit in 34 obese patients. Patients were divided according to preoperative weight loss, and peripheral blood mononuclear cells were compared using flowcytometry. The Δpreoperative excess weight loss [Δpre-EWL: pre-EWL (%)/period of preoperative weight loss (days)] showed negative correlations with total and subcutaneous fat area (*P* = .02, *r* = −0.41, *P* = .02, *r* = −0.42 respectively). The Δpre-EWL and Δpreoperative total weight loss (Δpre-TWL) were negatively correlated with white blood cell count, lymphocyte count, and C-reactive protein (CRP) levels at the initial visit (Δpre-EWL; *P* = .02, *r* = −0.37, *P* = .01, *r* = −0.41, *P* = .008, *r* = −0.45, Δpre-TWL; *P* = .01, *r* = −0.40, *P* = .01, *r* = −0.42, *P* = .01, *r* = −0.42, respectively). Multivariate regression modeling showed that both Δpre-EWL and Δpre-TWL were significantly associated with lymphocyte count (Δpre-EWL; *P* = .01, Δpre-TWL; *P* = .01). A comparison between the high (Δ pre-EWL > 0.098) and low weight loss group (Δ pre-EWL < 0.098) demonstrated a significant difference in the expression of the activation marker CD69 on CD56^bright^ Natural killer (NK) cells (*P* = .01), whereas there was no difference in the frequency of T cells, Natural killer T cells, or NK cells. Additionally, high CRP levels were associated with CD69 expression in CD56^bright^ NK cells (*P* = .01, *R* = 0.57). Peripheral lymphocytes, especially CD69-positive CD56^bright^ NK cells, are involved in preoperative weight loss after bariatric surgery, and systemic inflammation may inhibit weight loss before surgery.

## 1. Introduction

Obesity causes low-grade subacute chronic inflammation via immune cells and cytokines in tissues and organs, such as the adipose tissue, liver, pancreas, as well as the vascular system. It has been reported that this chronic inflammation is associated with metabolic disorders such as insulin resistance, cardiovascular disease, and fatty liver disease.^[1,2]^ Obesity and metabolic disorders are closely related to inflammation and immunity, and the concept of immunometabolism has been previously established.^[3]^

White blood cell (WBC) count and C-reactive protein (CRP) level are commonly used and widely available tests as indicators of inflammation. Many studies have shown that obesity promotes an increase in WBC count;^[4–6]^ furthermore, CRP levels are significantly correlated with body weight (BW), body mass index (BMI), and fat mass.^[7,8]^ It has also been reported that both WBC count and CRP levels decrease in patients with obesity after bariatric surgery.^[4,8]^ Several investigators have reported that immune cells are involved in obesity; however, few studies have investigated the relationship between the immune system and preoperative weight loss in patients before bariatric surgery.

Although much attention has been given to macrophages, T cells, and Natural killer T (NKT) cells in obesity-associated systemic inflammation, the role of Natural killer (NK) cells in obesity remains unknown. NK cells are classified as type 1 innate lymphoid cells that play an important role in innate immunity through their rapid responses to infection or tumor formation.^[9,10]^ These effector cells are also significant in the clinical progression of autoimmune diseases with chronic inflammation.^[9]^ Therefore, in the present study, we investigated the role of NK cells in preoperative weight loss in patients with obesity.

For patients with morbid obesity, bariatric surgery has proven to be more effective than other medical treatments in improving and resolving medical complications and their risks in addition to sustained long-term weight loss. The importance of preoperative weight loss as a requirement for bariatric surgery has long been debated. Although current clinical guidelines from the American Society for Metabolic and Bariatric Surgery do not recommend preoperative weight loss because there is no scientific evidence of its efficacy,^[11]^ many institutions provide preoperative medical weight management. One reason for this is the induction of behavior change; setting a goal for weight loss before surgery can promote behavior change in patients. Preoperative weight loss is an implied surrogate for intrinsic motivation in some practices.^[12]^ Additionally, weight loss can reduce liver volume. A larger liver increases the surgical difficulty because of the reduced intra-abdominal space and reduced freedom of surgical movement in the surgery.^[13]^ Based on the significance of these factors, we consider preoperative weight loss as the modality for preoperative risk reduction prior to bariatric surgery and mandate preoperative weight loss of 5% of initial weight before allowing patients to undergo surgery. However, there were individual differences in the period to lose 5% of initial weight before bariatric surgery. Hence, we conducted a study to investigate the association between preoperative weight loss and immune system status before bariatric surgery.

## 2. Methods

### 2.1. Study design, setting, and participants

Thirty-four patients (19 males and 15 females) who underwent laparoscopic sleeve gastrectomy (LSG) after preoperative weight loss for morbid obesity at Hiroshima University Hospital in Japan between March 2015 and July 2021 were included in the study. All patients satisfied the following criteria for LSG established by Japanese insurance practice guidelines: morbid obesity (BMI > 35 kg/m^2^), between 18 and 65 years of age, and insufficient control of ≥ 1 comorbidity by medical treatment for > 6 months (type 2 diabetes mellitus, hypertension, hyperlipidemia, or obstructive sleep apnea syndrome). The exclusion criteria were as follows: secondary obesity due to pituitary or endocrine disorder, poorly controlled mental disorder or alcoholism, and failure to lose 5% of the preoperative BW after 6 months of nutritional intervention with a formula diet (Mirodiet, Sunny Health Co. Ltd., Tokyo, Japan) taken at least once a day.

This study was approved by the Institutional Review Board of Hiroshima University Hospital (E611), and the study protocol conformed to the provisions of the Declaration of Helsinki in 1995 (as revised in Brazil 2013).^[14]^ Written informed consent was obtained from all patients at the initial visit.

The medical records of participants were reviewed for age, sex, type 2 diabetes mellitus, hypertension, hyperlipidemia, BW, BMI, weight loss, time to surgery, WBC, neutrophil, lymphocyte, and monocyte counts, and CRP level; and the visceral and subcutaneous fat areas (VFA and SFA, respectively) were determined by fat scan at the time of initial examination. Anthropometric measurements were recorded at the following time points: initial visit before surgery, day of surgery, and 12 months after surgery. The percentage weight change at each time point was defined as follows:^[15]^

1. Preoperative excess weight loss (EWL) (pre-EWL) (%): [(initial weight (kg) – weight at surgery (kg))/(initial weight (kg) – ideal weight (based on a BMI of 22 kg/m^2^) (kg))] × 100.
2. Preoperative total weight loss (TWL) (pre-TWL) (%): [(initial weight (kg) – weight at surgery (kg))/initial weight (kg)} × 100.
3. Δ pre-EWL (%): pre-EWL (%)/period of preoperative weight loss (days)
4. Δpre-TWL (%): pre-TWL (%)/period of preoperative weight loss (days)
5. Weight loss 12 months after surgery (%): [(weight on the day of surgery) – (weight at 12 months post-surgery)/(weight on the day of surgery)] ×100.

### 2.2. Isolation of PBMCs

Of the 34 patients who underwent LSG, 23 patients who consented to the collection of additional blood samples at the time of LSG were included in the analysis. Mononuclear cells were extracted from peripheral blood immediately after blood sampling. The blood specimens were diluted with phosphate-buffered saline, and peripheral blood mononuclear cells (PBMCs) were separated by standard density gradient centrifugation using Lympholyte®-H cell separation media, Human (Cedarlane Laboratories Ltd., Ontario, Canada) at 1500 rpm for 30 minutes. After the collection of PBMCs at interphase and washing with phosphate-buffered saline, the cell numbers were determined.

### 2.3. Antibodies and flow cytometric analysis

Flow cytometry was performed using a FACS Canto II (BD Biosciences, Mountain View, CA) and the results were analyzed using FlowJo software (Tree Star Software, San Carlos, CA). The PBMCs were stained immediately after purification with the following monoclonal antibodies: fluorescein isothiocyanate-conjugated anti-CD56, allophycocyanin-conjugated anti-CD3, phycoerythrin-conjugated anti-CD 69 (all from BD Pharmingen, San Diego, CA). The PBMCs were then incubated with antibodies for 30 minutes at 4°C in the dark. Dead cells were excluded from the analysis using 7-AAD (7-amino-actinomycin D, BD Pharmingen) or propidium iodide staining. Because of limited cell numbers, all surface markers could not be measured in every sample. In this analysis, T cells were defined as CD56^-^ CD3^+^ cells, NK cells as CD56^+^ CD3^-^ cells, and NKT cells as CD56^+^ CD3^+^cells (for the gating strategy see Figure S1, Supplemental Digital Content, http://links.lww.com/MD/J704).^[16]^

### 2.4. Statistical analysis

Data are presented as the median (interquartile range). The weight loss effect and immune cells in obese patients were analyzed using linear regression analysis. Comparisons of continuous variables were made using an unpaired Student *t* test. All statistical analyses were performed using statistical software JMP version 14 (SAS Institute, Cary, NC). A *P* value of < .05 was considered statistically significant.

## 3. Results

A total of 34 patients who had achieved preoperative weight loss were included in the study, of whom 44.1% were female, and the median age was 48 years (Table 1). Preoperatively, diabetes mellitus was identified in 20 patients (58.8%), hypertension in 24 patients (70.6%), and hyperlipidemia in 12 patients (35.3%). The median time from the initial visit to surgery was 151 (125–212) days. The median initial BW was 120.6 (103.5–138) kg, and BMI was 45.2 (38.4–46.7) kg/m^2^. The median preoperative BW was 108.5 (94.6–126.9) kg, and BMI was 39.8 (35.9–43.7) kg/m^2^. The median preoperative weight loss was 9.1 (5.45–13.2) kg. The median pre-EWL and pre-TWL were 13.29% (11.39–24.84%) and 7.06% (5.01–11.03%), respectively. Since there were individual differences in the preoperative weight loss period, preoperative weight loss was divided by the weight loss period to obtain the median Δ pre-EWL and Δ pre-TWL. The Δpre-EWL was 0.098% (0.075–0.148%) and Δpre-TWL was 0.049% (0.039–0.069%).

**Table 1:** Patient characteristics.

	Total (n = 34)
Age, yr	48 [41–52]
Female	15 (44.1%)
Diabetes mellitus II	20 (58.8%)
Hypertension	24 (70.6%)
Hyperlipidaemia	12 (35.3%)
Initial BW, kg	120.6 [103.5–138]
Initial BMI, kg/m^2^	45.2 [38.4–46.7]
Preoperative BW, kg	108.5 [94.6–126.9]
Preoperative BMI, kg/m^2^	39.8 [35.9–43.7]
Preoperative weight loss, kg	9.1 [5.45–13.2]
Time to surgery, d	151 [125–212]
Pre EWL, %	13.29 [11.39–24.84]
Pre TWL, %	7.06 [5.01–11.03]
Δpre EWL, %	0.098 [0.075–0.148]
Δpre TWL, %	0.049 [0.039–0.069]

Values are shown as median (interquartile range [IQR]) and number (percentage).

BMI = body mass index, BW = body weight, Pre EWL = preoperative excess weight loss, Pre TWL = preoperative total weight loss, Δpre-EWL = Δpreoperative excess weight loss, Δpre-TWL = Δpreoperative total weight loss.

**Figure 1.**
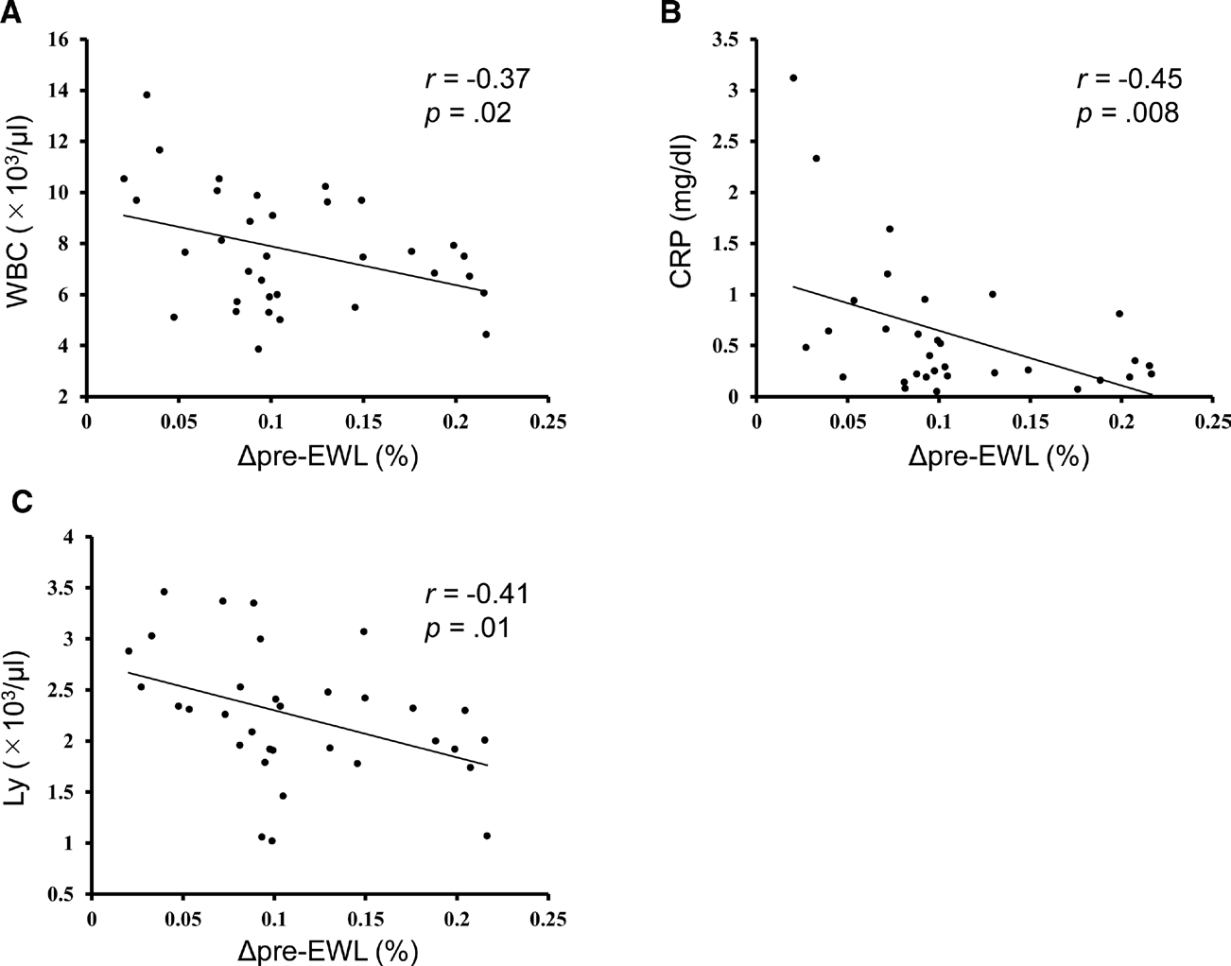
Correlation between clinical parameters and Δpreoperative excess weight loss. (A) Correlation between WBC count and Δpre-EWL. (B) Correlation between CRP level and Δpre-EWL. (C) Correlation between lymphocyte count and Δpre-EWL. CRP = C-reactive protein, Ly = Lymphocyte, Δpre-EWL = Δpreoperative excess weight loss, WBC = white blood cell.

Table 2 shows the factors associated with pre-EWL and pre-TWL. The pre-EWL was not associated with any of the factors, while the pre-TWL was positively correlated with BMI at the time of the initial visit, but not with the other factors, such as WBC count, lymphocyte count, or fat area. Table 3 shows the factors associated with Δpre-EWL and Δpre-TWL. The Δpre-EWL showed negative correlations with total fat area (TFA) and SFA (TFA: *r* = −0.41, *P* = .02; SFA: *r* = −0.4244, and *P* = .02), and was not significantly associated with BMI and VFA. Additionally, it was negatively and significantly associated with initial WBC count and CRP levels (WBC count: *r* = −0.37, *P* = .02; CRP levels: *r* = −0.45, *P* = .008) (Fig. 1A and B). The Δpre-TWL also showed a significant negative correlation with initial WBC count and CRP levels (WBC count: *r* = −0.40, *P* = .01; CRP levels: *r* = −0.42, *P* = .01). Furthermore, only lymphocyte count was associated with Δ pre-EWL and Δ pre-TWL, while neutrophil and monocyte counts were not (Δ pre-EWL: *r* = −0.41, *P* = .01; Δ pre-TWL: *r* = −0.42, *P* = .01) (Fig. 1C). Multivariate regression modeling showed that Δpre-EWL was associated with SFA and lymphocyte count (*R*^2^ = 0.24; *P* = .01); while Δpre-TWL was associated with lymphocyte count only (*R*^2^ = 0.15; *P* = .01). Both Δpre-EWL and Δpre-TWL were associated with initial lymphocyte count, and patients with high lymphocyte counts had lower preoperative weight loss (Table 4).

**Table 2:** Correlations between preoperative excess weight loss, preoperative total weight loss and selected variables.

Parameter	pre-EWL		pre-TWL	
	*r*	*P* value	*r*	*P* value
Initial BMI (kg/m^2^)	0.04	.82	0.33	.05
Total fat area (cm^2^)	−0.28	.13	−0.02	.89
Subcutaneous fat area (cm^2^)	−0.25	.18	0.01	.95
Visceral fat area (cm^2^)	−0.24	.20	−0.07	.70
Initial WBC count	−0.23	.18	−0.25	.14
Initial neutrophil counts	−0.20	.25	−0.24	.16
Initial lymphocyte counts	−0.24	.16	−0.24	.17
Initial monocyte counts	−0.25	.15	−0.29	.10
Initial CRP levels (mg/dL)	−0.25	.16	−0.21	.23

BMI = body mass index, CRP = C-reactive protein, pre-EWL = preoperative excess weight loss, pre-TWL = preoperative total weight loss, *r* = Pearson’s correlation coefficient, WBC = white blood cells.

**Table 3:** Correlations between Δpreoperative excess weight loss, Δpreoperative total weight loss and selected variables.

Parameter	Δpre-EWL		Δpre-TWL	
	*r*	*P* value	*r*	*P* value
Initial BMI (kg/m^2^)	−0.14	.42	0.20	.24
Total fat area (cm^2^)	−0.41	.02*	−0.13	.49
Subcutaneous fat area (cm^2^)	−0.42	.02*	−0.14	.44
Visceral fat area (cm^2^)	−0.26	.17	−0.07	.70
Initial WBC count	−0.37	.02*	−0.40	.01*
Initial neutrophil counts	−0.29	.09	−0.33	.05
Initial lymphocyte counts	−0.41	.01*	−0.42	.01*
Initial monocyte counts	−0.21	.23	−0.27	.12
Initial CRP level (mg/dL)	−0.45	.008**	−0.42	.01*

BMI = body mass index, CRP = C-reactive protein, Δpre-EWL = Δpreoperative excess weight loss, Δpre-TWL = Δpreoperative total weight loss, *r* = Pearson’s correlation coefficient, WBC = white blood cells.

* *P* < .05.

** *P* < .01.

**Table 4:** Multivariate regression analysis with preoperative weight loss.

	Adjusted *R*^2^	Standardized β	*T*	*P* value
Δpre-EWL	0.24			.01*
Subcutaneous fat area (cm^2^)		−0.37	−2.24	.03*
Initial lymphocyte counts		−0.37	−2.19	.04*
Δpre-TWL	0.15			.01*
Initial lymphocyte counts		−0.43	−2.62	.01*

Δpre-EWL = Δpreoperative excess weight loss, Δpre-TWL = Δpreoperative total weight loss.

* *P* < .05.

**Figure 2.**
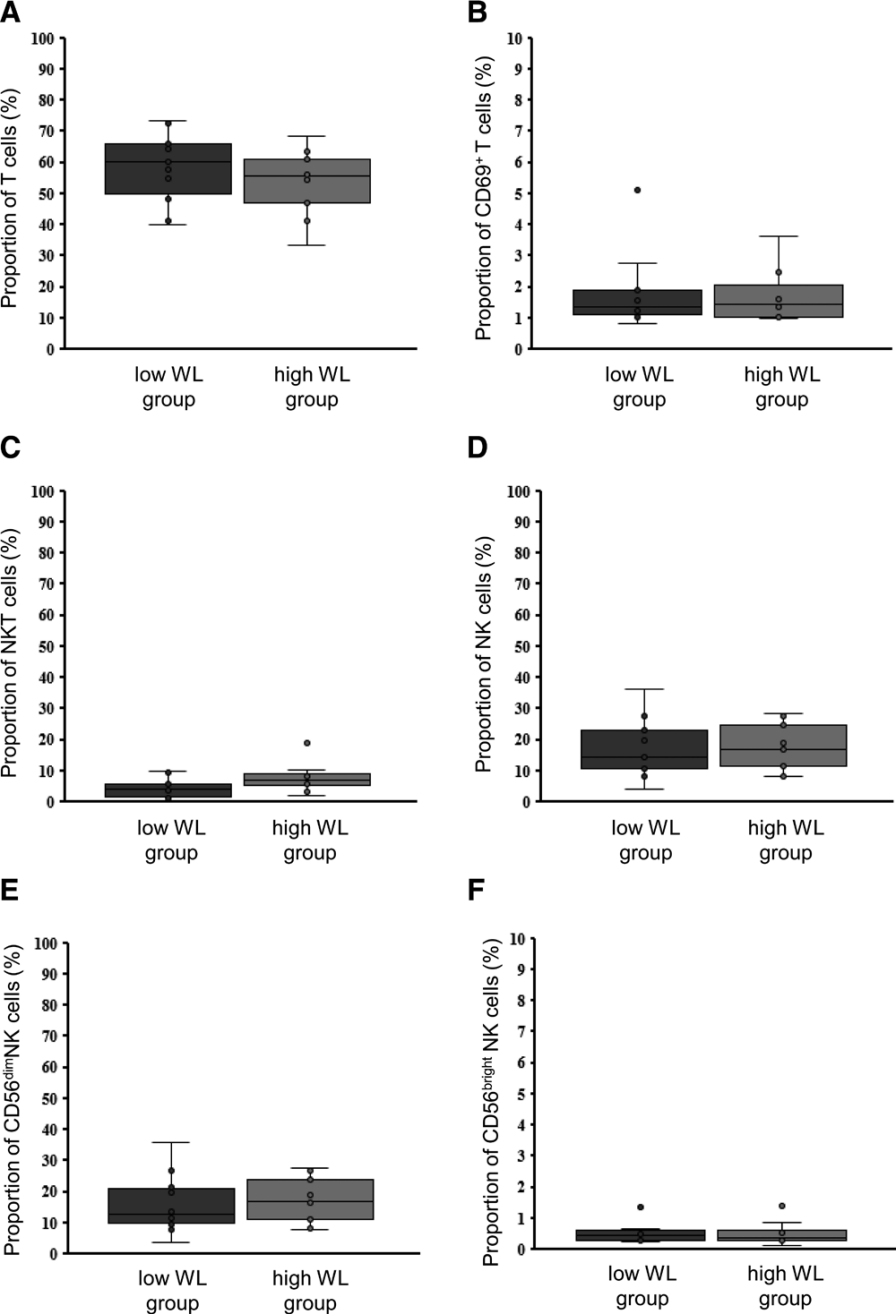
Association preoperative body weight loss and immune cells. Flow cytometric analysis of T cells, NKT cells, NK cells and subsets of each cell in peripheral blood mononuclear cells (PBMCs) isolated from the low weight loss (WL) group (Δpre-EWL < 0.098; low WL) and high weight loss group (Δpre-EWL > 0.098; high WL). (Frequency of T cells (A), NKT cells (C), NK cells (D) in PBMCs. The expression of CD69 on T cells (B). Expression of CD56^dim^ (E) and CD56^bright^ (F) subset on NK cells. Data are shown as the median, 25th and 75th percentiles, and range. Student *t* test for unpaired data was used to evaluate the differences between low and high WL. NK cells = natural killer cells, NKT cells = natural killer T cells, WL = weight loss.

**Figure 3.**
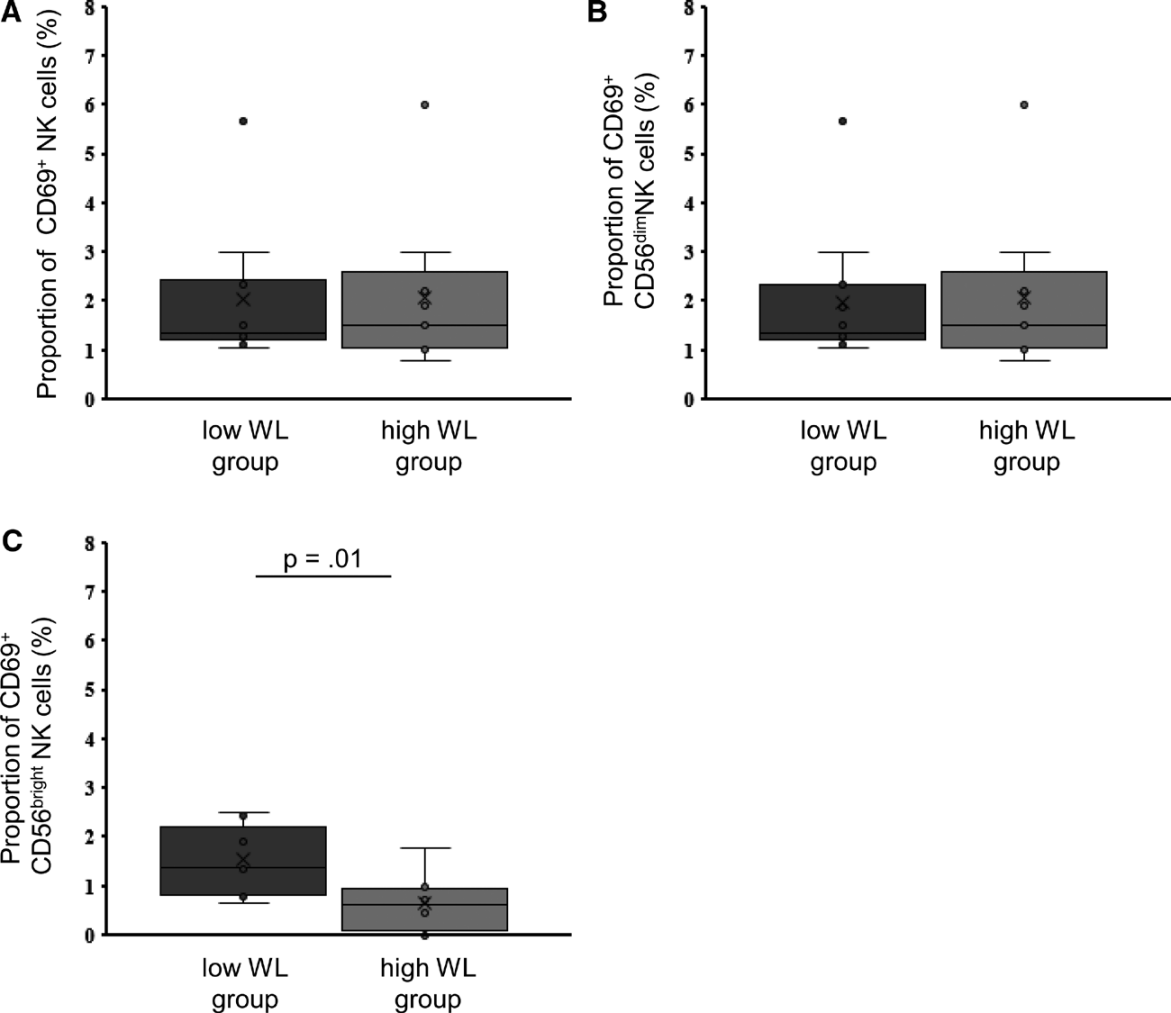
Association preoperative body weight loss and expression of CD69 on NK cells. Expression of CD69 on NK cells (A), CD56^dim^ NK cells (B) and CD56^bright^ NK cells (C). NK cells = natural killer cells, WL = weight loss.

**Figure 4.**
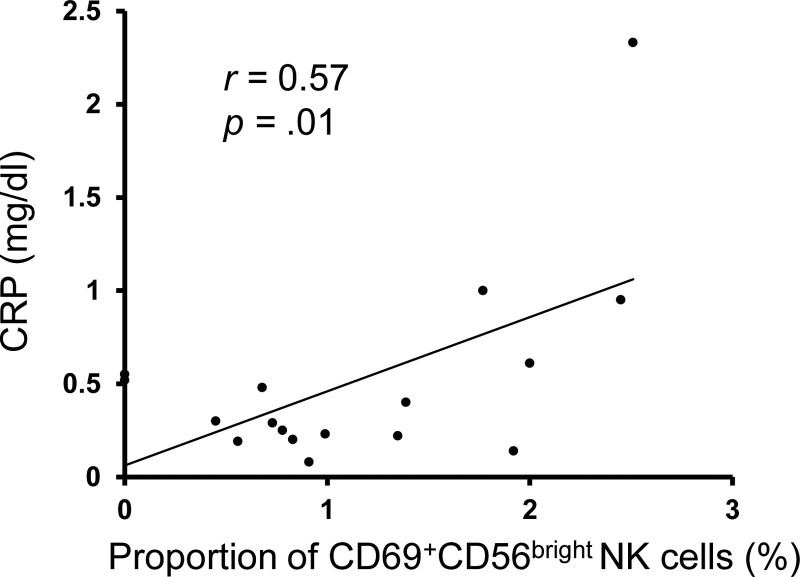
Correlation between CRP levels and CD69 expression on CD56^bright^ NK cells. CRP = C-reactive protein, NK cells = natural killer cells.

To determine whether the profile of immune cells in the peripheral blood was associated with preoperative weight loss, FACS analysis was performed on PBMCs isolated from patients before surgery (Figure S1, Supplemental Digital Content, http://links.lww.com/MD/J704). Patients were divided based on the median Δpre-EWL; those with Δpre-EWL > 0.098 were defined as the high weight loss group (n = 11), and those with Δ pre-EWL < 0.098 were defined as the low weight loss group (n = 12). The frequencies of T cells (CD56^−^ CD3^+^), NKT cells (CD56^+^ CD3^+^), and NK cells (CD56^+^ CD3^−^) did not differ between the 2 study groups (Fig. 2A, C, and D). There was no difference in the expression of CD69, an activation marker, on T cells between the 2 groups (Fig. 2B). The NK cells were subsequently divided into CD56^bright^ or CD56^dim^ subsets based on the expression level of CD56. The frequency of CD56^bright^ and CD56^dim^ subsets among total NK cells did not differ between the 2 groups (Fig. 2E and F). There were no differences in the expression of CD69 on NK cells and CD56^dim^ NK cells between the 2 groups, whereas the CD69 expression on CD56^bright^ NK cells was significantly higher in the low weight loss group (*P* = .01) (Fig. 3). Furthermore, the CD69 expression on CD56^bright^ NK cells had a significant positive correlation with CRP levels (Fig. 4).

Consequently, we investigated the relationship between chronic inflammation and postoperative weight loss at 12 months (n = 32). Postoperative EWL and TWL were not associated with WBC count, CRP level, lymphocyte count, neutrophil count, or monocyte count (Figure S2, Supplemental Digital Content, http://links.lww.com/MD/J705) at 12 months. Similarly, pre-EWL and Δpre-EWL were not correlated with postoperative EWL at 12 months (Figure S3, Supplemental Digital Content, http://links.lww.com/MD/J706). The pre-TWL and Δ pre-TWL were not associated with postoperative EWL (data not shown).

## 4. Discussion

In this study, preoperative weight loss was more dependent on SFA than VFA (Tables 3 and 4). This result was consistent with a previous report showing that weight loss after bariatric surgery was negatively correlated with preoperative SFA and not VFA in Japanese patients with obesity.^[17]^ Our results show that preoperative weight loss was associated with increased initial WBC count, lymphocyte count, and CRP levels, indicating that chronic inflammation inhibits medical weight management before surgery (Fig. 1). Multivariate regression analysis showed that weight loss was most strongly associated with the initial lymphocyte count (Table 4). Lymphocytes are immune cells that are a subset of WBCs and include T cells, B cells, NK cells, and NKT cells. Our study did not reveal a significant correlation between Δpre-EWL/Δpre-TWL and each proportion of T cells, NKT cells, and NK cells (Figure S4, Supplemental Digital Content, http://links.lww.com/MD/J707); hence, the patients were divided into high and low weight loss groups for further comparison. Since we found that a higher CRP level resulted in a lower Δpre-EWL (Fig. 1B), we investigated the expression CD69 as an activation marker on each immune cell in the high weight loss and low weight loss groups (Fig. 3).

Neither the proportion of NK cells in lymphocytes nor the proportion of CD56^bright^ and CD56^dim^ NK cells among the total NK cells affected preoperative weight loss, whereas the expression of CD69 on CD56^bright^ NK cells did affect preoperative weight loss (Fig. 3). The CD56^dim^ NK cell subset is the major NK cell subpopulation in peripheral blood and has been described as a more naturally cytotoxic subset.^[18]^ In contrast, CD56^bright^ NK cells mediate immunomodulatory effects by secreting abundant amounts of cytokines such as tumor necrosis factor-alpha (TNF-α) and interferon gamma but have low cytotoxicity. Bähr et al^[19]^ reported that the percentage of circulating CD56^bright^ NK cells was significantly increased in obesity compared to non-obese individuals and that there was a significant positive correlation between CD56^bright^ NK cells and BMI in participants with normal weight and those with obesity. Lynch et al^[20]^ reported that metabolically unhealthy obese patients expressed significantly higher CD69 in circulating NK cells than healthy patients with obesity. In the present study, CD69 expression on CD56^bright^ NK cells was significantly higher in the low weight loss group and was correlated with CRP level (Fig. 4), suggesting that an increased activated CD56^bright^ NK cell subset may substantially increase the production of cytokines such as TNF-α and interferon gamma, contributing to poor weight loss. Furthermore, cytokines are generally accepted to induce protein synthesis during acute inflammation in the liver and the resultant elevation of CRP regulate the function of NK cells.^[21]^ Several cytokines, including IL-2, IL-15, and IL-21, have been shown to enhance CD69 expression of NK cells, resulting in the augmentation of NK-cell activity.^[22–24]^ In the obese state, the production of a plethora of cytokines by macrophage and other immune cells lead to systemic inflammation, and these may lead to increased CD69 expression on NK cells.

It is important to note that CD69 has not only been identified as an early activation marker but is also known to be associated with tissue residency by suppressing the surface expression of sphingosine-1-phosphate receptor 1.^[25]^ It has also been reported that CD69 is rarely expressed or absent on circulating NK cells in healthy donors and that CD69 expression may be tissue-specific.^[20,26]^ In the obese state, immune cells in adipose tissue are greatly altered, and the CD69 expression rate on NK cells in the visceral adipose tissue (VAT) of obese mice was higher than that of NK cells in the spleen.^[27]^ From these findings we can infer that there is a possibility that circulating NK cells that contribute to chronic inflammation such as CD69-positive CD56^bright^ NK cells may migrate into the circulation from adipose tissue in the obese state. In addition, CD56^bright^ NK cells expressing tissue-specific CD69 are involved in preoperative weight loss, suggesting that local inflammation in the VAT may spread systemically and affect weight loss. Further studies investigating the phenotype and function of VAT NK cells in obesity are required to clarify the involvement of the immune system in metabolic disorders and in weight loss in obesity. Hence, we are now analyzing the immune cells of VAT from patients who have undergone LSG to concretize the functional importance of NK cell populations.

In addition to NK cells, other lymphocytes should also be considered in immunometabolism. Both T cells and B cells facilitate adaptive immunity, and T cells are further differentiated into helper, regulatory, cytotoxic, or memory T-cells. McLaughlin et al^[28]^ have previously demonstrated that T-helper (Th)-1 cell frequency in VAT correlated directly, whereas Th2 frequency in VAT was inversely correlated with plasma CRP. Additionally, Th2 in peripheral blood has also been inversely associated with systemic insulin resistance. In the present study, we were unable to elucidate the involvement of peripheral T cells and CD69^+^ T cells in weight loss before surgery (Fig. 2), and thus future analysis should focus on lymphocytes of VAT. It has been reported that DNA methylation levels in B cells are increased in patients with obesity when compared to lean individuals and positively correlated with insulin resistance.^[29]^ The role of NKT cells in the development of metabolic disorders is controversial; some studies have reported that the deletion of NKT cells improves insulin resistance in obesity, while other studies have shown the opposite.^[30–33]^ Because of the limited number of cells, we could not perform comprehensive flow cytometry for NKT cell and B cell subsets.

The relationship between postoperative weight loss and immune cells in bariatric surgery has been previously reported in the literature, with the preoperative neutrophil-to-lymphocyte ratio being a prognostic factor for long-term weight loss after bariatric surgery.^[34]^ On the contrary, there have been no reports on the relationship between preoperative medical weight management and immunity. In this study, we used Δpre-EWL and Δpre-TWL divided by the preoperative period instead of pre-EWL and pre-TWL to eliminate the effect of the preoperative weight loss period, which varies among individuals, and to analyze the relationship between preoperative weight loss and immunity. Our results showed that patients with high WBC count, lymphocyte count, and CRP level were less likely to lose weight before surgery, meaning that patients with high preoperative inflammation should receive enhanced preoperative medical weight management and bariatric surgery may be preceded even if preoperative weight loss does not reach 5%.

We acknowledge several limitations of this study. First, we were unable to reveal the concrete value of the peripheral lymphocyte count as a predictor of preoperative weight loss due to the lack of general criteria for sufficient weight loss before surgery. Second, our cohort was too small to show a difference in the subset of lymphocytes between the high- and low-weight loss groups.

## 5. Conclusion

This study is the first to demonstrate that lymphocyte count significantly affects preoperative weight loss in bariatric surgery and that CD69-positive CD56^bright^ NK cells are involved in preoperative weight loss. This issue indicates that systemic inflammation may inhibit weight loss. Prevention of systemic inflammation due to CD69-positive CD56^bright^ NK cells may represent a promising approach for obesity treatment.

## Acknowledgments

The authors thank Minoru Hattori for supporting statistical expertise. We would like to thank Editage (www.editage.jp) for English language editing.

## Author contributions

**Conceptualization:** Yoshihiro Saeki, Hideki Ohdan.

**Data curation:** Emi Chikuie, Kazuaki Tanabe, Hiroshi Ota.

**Formal analysis:** Emi Chikuie, Yuka Tanaka.

**Funding acquisition:** Yoshihiro Saeki.

**Investigation:** Emi Chikuie.

**Methodology:** Emi Chikuie, Yoshihiro Saeki, Yuka Tanaka.

**Project administration:** Yoshihiro Saeki.

**Resources:** Emi Chikuie, Yoshihiro Saeki.

**Software:** Emi Chikuie.

**Supervision:** Yoshihiro Saeki, Hideki Ohdan.

**Validation:** Emi Chikuie.

**Visualization:** Emi Chikuie.

**Writing – original draft:** Emi Chikuie, Yoshihiro Saeki.

**Writing – review & editing:** Yoshihiro Saeki, Kazuaki Tanabe, Hiroshi Ota, Yuka Tanaka, Hideki Ohdan.

## Supplementary Material
