# Supplementary material for: The involvement of circulating CD69+ CD56bright natural killer cells in weight loss before bariatric surgery: A retrospective cohort study
Source: Medicine (Baltimore). 2023 Oct 13;102(41):e34999. doi: 10.1097/MD.0000000000034999 (PMC10578777; doi:10.1097/MD.0000000000034999)
Supplement: Supplementary file 1 [file medi-102-e34999-s001.pptx]

## Slide 1
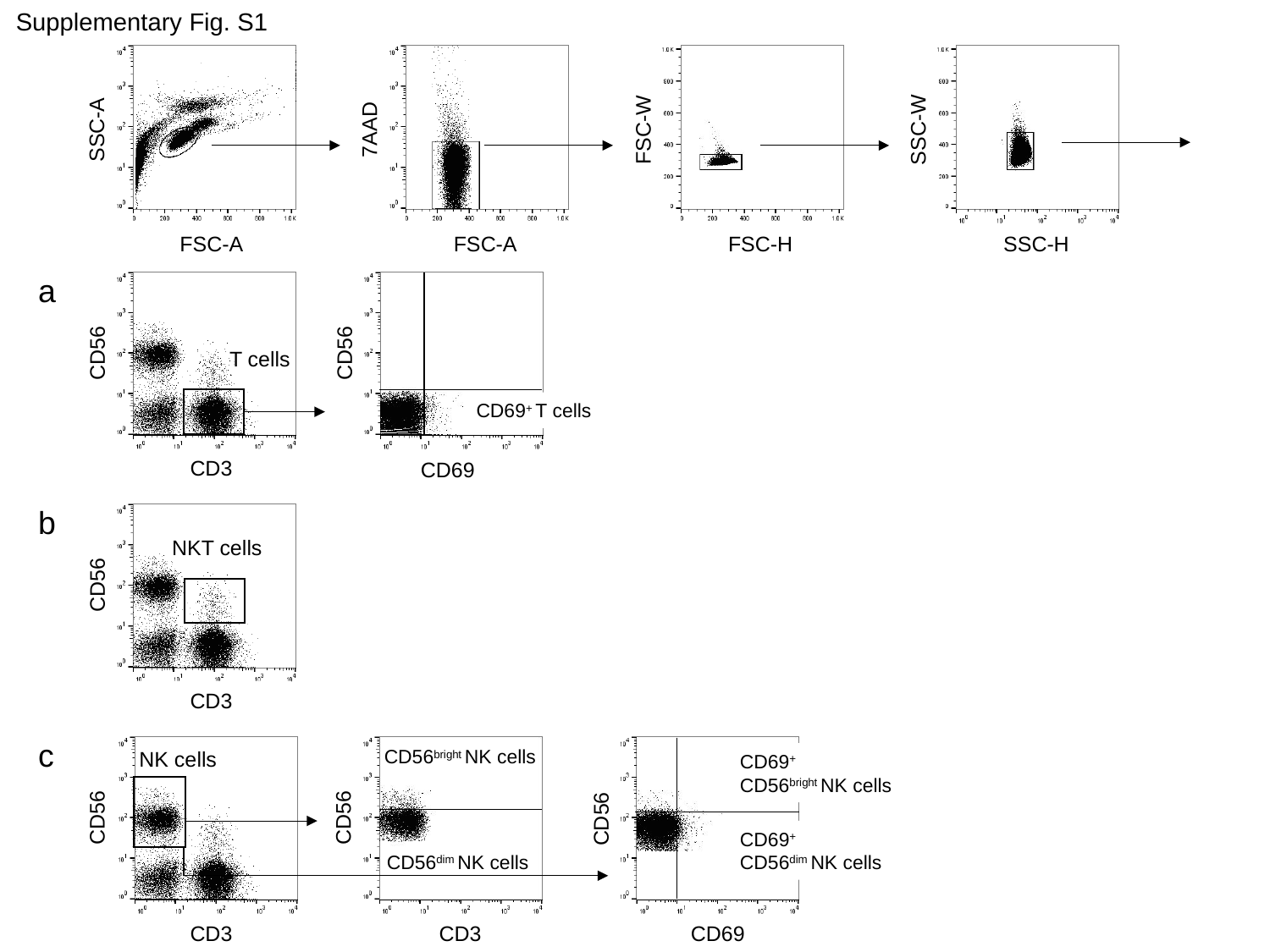

Supplementary Fig. S1
SSC-A
7AAD
FSC-W
SSC-W
FSC-A
FSC-A
FSC-H
SSC-H
a
CD56
CD56
T cells
CD69+ T cells
CD3
CD69
b
NKT cells
CD56
CD3
c
CD56bright NK cells
NK cells
CD69+
CD56bright NK cells
CD56
CD56
CD56
CD69+ CD56dim NK cells
CD56dim NK cells
CD3
CD3
CD69
