# Supplementary material for: The involvement of circulating CD69+ CD56bright natural killer cells in weight loss before bariatric surgery: A retrospective cohort study
Source: Medicine (Baltimore). 2023 Oct 13;102(41):e34999. doi: 10.1097/MD.0000000000034999 (PMC10578777; doi:10.1097/MD.0000000000034999)
Supplement: Supplementary file 2 [file medi-102-e34999-s002.pptx]

## Slide 1
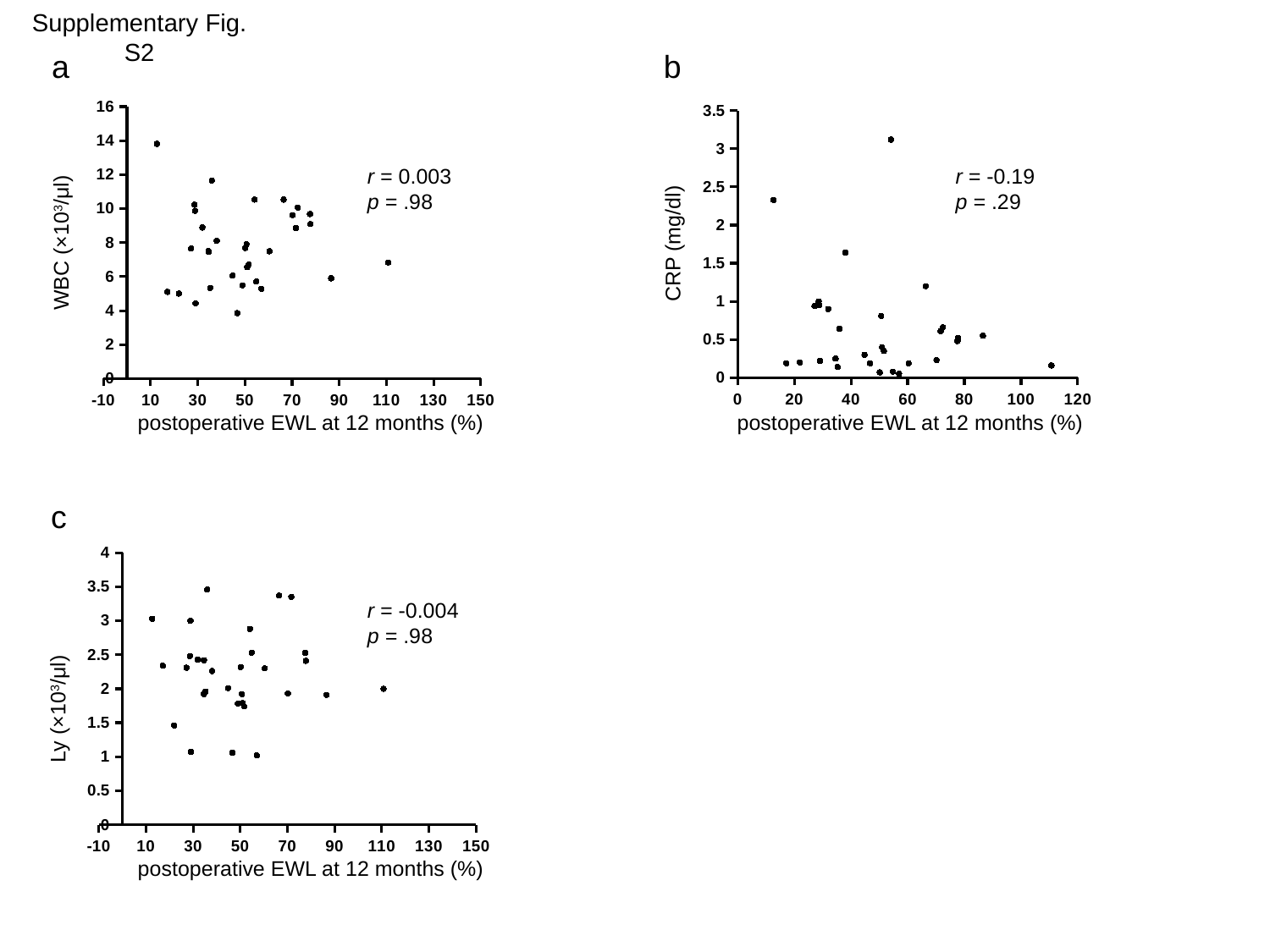

Supplementary Fig. S2
a
b
### Chart
| Category | |
|---|---|
### Chart
| Category | |
|---|---|r = -0.19
p = .29
r = 0.003
p = .98
WBC (×103/μl)
CRP (mg/dl)
 postoperative EWL at 12 months (%)
 postoperative EWL at 12 months (%)
c
### Chart
| Category | |
|---|---|r = -0.004
p = .98
Ly (×103/μl)
 postoperative EWL at 12 months (%)
