# Supplementary material for: The involvement of circulating CD69+ CD56bright natural killer cells in weight loss before bariatric surgery: A retrospective cohort study
Source: Medicine (Baltimore). 2023 Oct 13;102(41):e34999. doi: 10.1097/MD.0000000000034999 (PMC10578777; doi:10.1097/MD.0000000000034999)
Supplement: Supplementary file 4 [file medi-102-e34999-s004.pptx]

## Slide 1
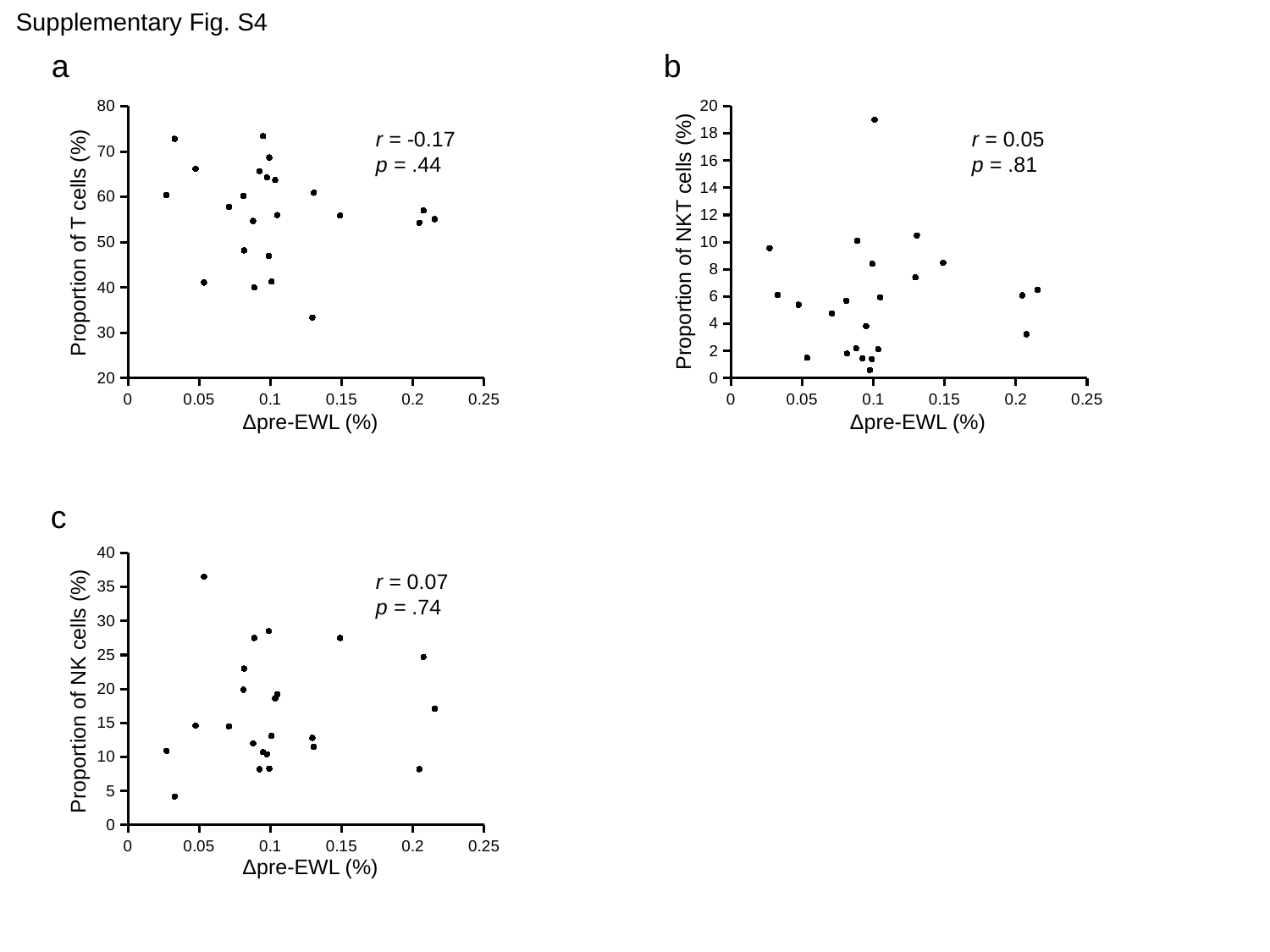

Supplementary Fig. S4
a
b
### Chart
| Category | |
|---|---|
### Chart
| Category | |
|---|---|r = -0.17
p = .44
r = 0.05
p = .81
Proportion of NKT cells (%)
Proportion of T cells (%)
Δpre-EWL (%)
Δpre-EWL (%)
c
### Chart
| Category | |
|---|---|r = 0.07
p = .74
Proportion of NK cells (%)
Δpre-EWL (%)
